# Atrial fibrillation designation with micro-Raman spectroscopy and scanning acoustic microscope

**DOI:** 10.1038/s41598-022-10380-z

**Published:** 2022-04-19

**Authors:** Ugur Parlatan, Seyma Parlatan, Kubra Sen, Ibrahim Kecoglu, Mustafa Ozer Ulukan, Atalay Karakaya, Korhan Erkanli, Halil Turkoglu, Murat Ugurlucan, Mehmet Burcin Unlu, Bukem Tanoren

**Affiliations:** 1 Department of Physics, Bogazici University, Istanbul, 34342 Turkey; 2 Vocational School of Health Services, Istinye University, Istanbul, 34020 Turkey; 3 Department of Cardiovascular Surgery, Istanbul Medipol University, Istanbul, 34214 Turkey; 4 Global Station for Quantum Medical Science and Engineering, Global Institution for Collaborative Research and Education (GI-CoRE), Hokkaido University, Sapporo, Japan; 5 Department of Natural Sciences, Acıbadem University, Istanbul, 34684 Turkey

**Keywords:** Cardiology, Astronomy and planetary science, Chemistry, Optics and photonics, Physics

## Abstract

Atrial fibrillation (AF) is diagnosed with the electrocardiogram, which is the gold standard in clinics. However, sufficient arrhythmia monitoring takes a long time, and many of the tests are made in only a few seconds, which can lead arrhythmia to be missed. Here, we propose a combined method to detect the effects of AF on atrial tissue. We characterize tissues obtained from patients with or without AF by scanning acoustic microscopy (SAM) and by Raman spectroscopy (RS) to construct a mechano-chemical profile. We classify the Raman spectral measurements of the tissue samples with an unsupervised clustering method, k-means and compare their chemical properties. Besides, we utilize scanning acoustic microscopy to compare and determine differences in acoustic impedance maps of the groups. We compared the clinical outcomes with our findings using a neural network classification for Raman measurements and ANOVA for SAM measurements. Consequently, we show that the stiffness profiles of the tissues, corresponding to the patients with chronic AF, without AF or who experienced postoperative AF, are in agreement with the lipid-collagen profiles obtained by the Raman spectral characterization.

Coronary artery bypass surgery, which redirects blood through a healthy vessel taken from the leg, arm, or chest, is the surgical technique applied to patients with blocked or partially blocked arteries in their hearts. Approximately in half of the patients after coronary artery bypass surgery, atrial fibrillation (AF) is developed. Even though the exact cause of AF is not known, it is described to be mostly rapid and irregular heart rate, which increases the risk of heart complications such as stroke. AF was associated with fibrotic content of fatty infiltrations in the left atria of AF sheep^1^. In patients with AF, blood clots can be developed and these may circulate within the body and then cause severe damages to specific organs. Therefore, possible causes of AF are widely investigated by the scientists.

## Introduction

The gold standard in diagnosing AF is the visual investigation of the electrocardiograms that are evaluated by the doctor’s clinical experience^2^. Artificial intelligence brings a quantitative perspective to the diagnosis process^3,4^. In addition, the physical measurement of human tissues evaluated frequently, brings another perspective to quantify the diagnosis of a specific disease. Such efforts for AF using Raman spectroscopy have been proposed before^5^.

Molecular spectroscopy enables the non-invasive structure determination of tissues. Raman spectroscopy is a vibrational spectroscopic technique that enables monitoring chemical signatures of the molecules in a sample by measuring the inelastic scattering photons spreading away from the illuminated sample^6^. An electromagnetic wave’s interaction (collision) with a molecule in a material leads to a shift in the frequency when the scattering is inelastic. In addition to its widespread use in chemistry^7,8^, biology^9,10^ and materials science^11,12^, this spectroscopic technique has also shown a great potential in medical applications such as diagnosing cancers, cardiovascular diseases and dermatological diseases^13–15^.

Tissue characterization with Raman spectroscopy enables fast determination of disease states^16^ and pathological margins^17,18^ by decomposing the spectral tissue response and characterizing the macromolecular structure that mainly contains collagen, protein, and lipids. Machine learning and Raman spectroscopy have recently been an appropriate couple to detect small spectral changes in biological samples that may lead to a decision about the sample^19,20^. One of these methods is k-means clustering that is an unsupervised data analysis method^21,22^. Measurements can be grouped into several clusters with no previous information using k-means.

Scanning acoustic microscopy (SAM) is a technique that uses ultrasound signals to obtain the samples’ morphological and mechanical properties simultaneously. The speed of sound (SOS) through tissues^23–26^ or acoustic impedance of samples^27,28^ can be obtained by SAM. On the other hand, two major advantages of SAM are its high speed in obtaining the two-dimensional maps and instantaneous scanning of the specimen with no staining or specific preparation.

In this study, we utilized scanning acoustic microscopy with Raman spectroscopy to investigate the samples obtained from patients, who underwent coronary artery bypass surgery. We used our Raman spectra and SAM images to group the tissues by performing a k-means algorithm. Consistency in SAM and RS results proved the success in combining these techniques to obtain a mechano-chemical profile for atrial tissues.

## Results and discussion

In this study, 65 tissue samples (one tissue sample from each patient) were obtained from the auricle of the right atrium of patients and examined with both SAM and RS. There are 3 groups of patients, who are with chronic AF, who experienced postoperative AF and converted to sinus rhythm either medically or by cardioversion, and who did not experience rhythm disturbances preoperatively or after the operation during their hospital stay.

### Raman spectroscopy

**Figure 1:**
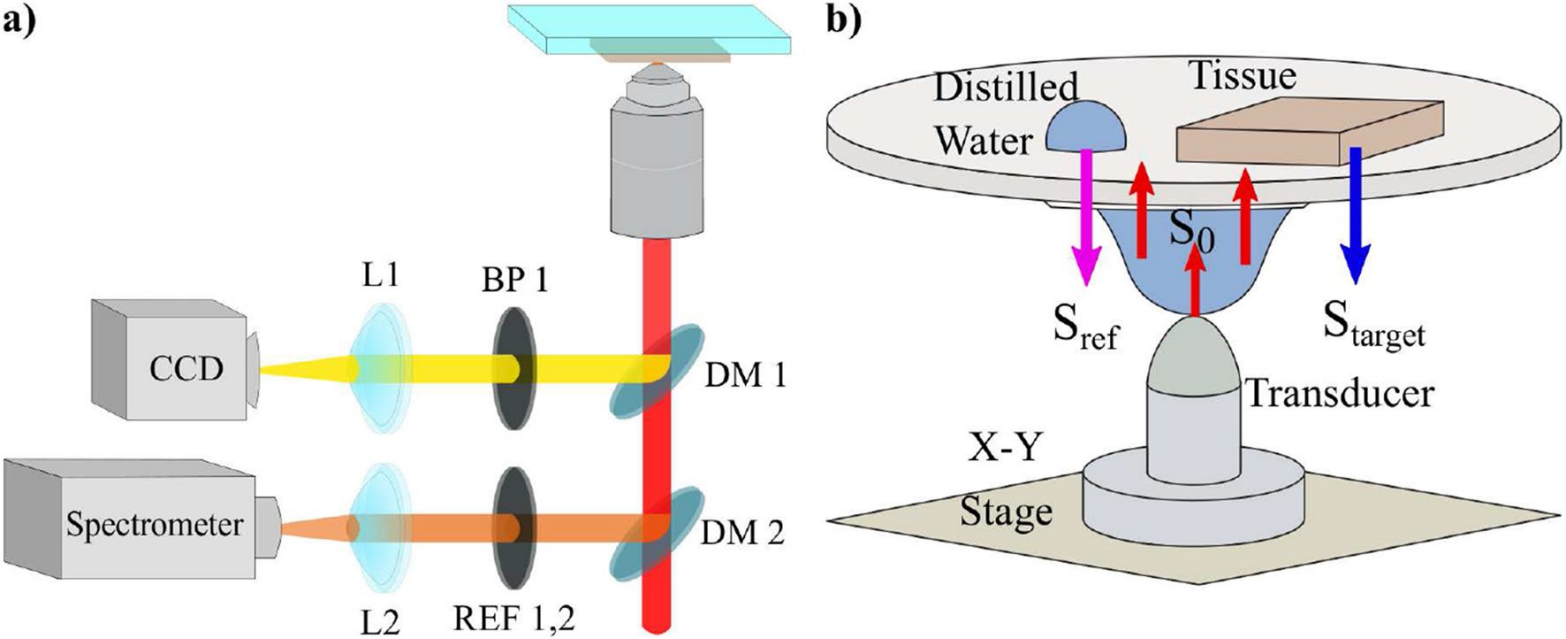
Experimental setups for (**a**) Raman spectroscopy and (**b**) scanning acoustic microscopy.

We scanned each tissue sample using the experimental setup shown in Fig. 1a. We acquired Raman spectra of tissues from 441 different sites for each sample, which corresponds to a scan area of 1 x 1 mm^2^. Thus, we obtained a sampling that roughly represents the heterogeneous structure of the sample. We obtained a total number of 30552 spectra from 65 tissue samples. To reduce the number of variables in the analysis, we applied a principal component analysis (PCA) on the dataset we constructed from all the spectra that were baseline corrected and vector normalized.

#### Unsupervised (k-means) clustering of the spectra

**Figure 2:**
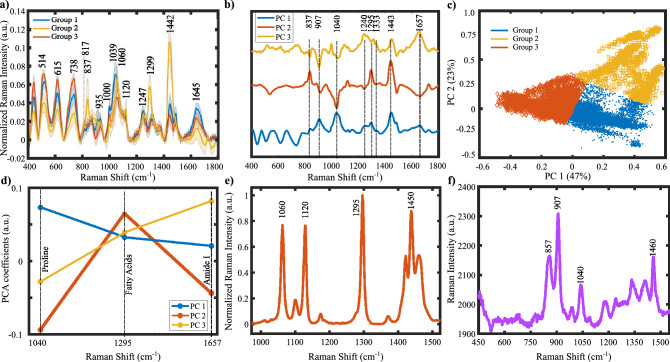
Raman spectroscopy-based classification of the measured tissue samples. (**a**) Comparison of group means. (**b**) PC loadings of the spectral distribution of the first three PCs whose TVEs are about 76%. We used these PCs in the k-means analysis. (**c**) Determination of group indices by using principal component analysis (PCA). PC1 (with total variance explained (TVE) of 47%) vs. PC2 (TVE of 23%) plot shows the Euclidean distance between the PC scores, which were further used in the k-means analysis for the determination of group identities. (**d**) Chemical comparison of PC loadings by means of their responses at particular band positions. Variations of peak positions at 1040, 1295 and 1657 (proline, fatty acids, and Amide I) were investigated. (**e**) Raman spectrum of Stearic Acid (Provided by FDM Raman organics library). (**f**) Raman spectrum of Proline measured in our lab.

AF can be mechanically characterized by giving indices to the stiffness levels of the tissues. Although we had this information, we considered this research problem as an unsupervised case from the molecular characterization view. We applied k-means clustering on the first 20 principal components that cover 91% of the total variance explained. We utilized the Calinski-Harabasz index to determine the optimal “k” value for k-means clustering and found the k value as three. In Fig. 2a, we showed the averages of the three groups obtained from k-means clustering. The spectra in groups still had significant variances since the grouping is mainly performed depending on the amount of particular chemical(s). However, averaging a group can help explore the data and understand which spectral components are dominant in each group. For example, the first group mainly consists of the contributions from collagen bands since the highest intensity components are at 915, 1000, 1040, 1247, 1450, and 1645 cm^-1^. On the other hand, the second group is dominated by fatty acid components since the sharp peaks at 1060, 1120, 1299, and 1450 cm^-1^ exist. Besides, the band at 1725 cm^-1^, which is related to ester bonds, is only apparent in this group. The third group has relatively strong band components from Amide III (1254, 1265) and Amide I (1645). The number of spectra in the second group corresponds to 5.23% of all measurements. Thus, the measurements in the first and the third group are more dominant when compared to group two.

#### Utilizing PC Spectra and Band Component Analysis to Obtain Chemical Information

In Fig. 2c, the PCA scores were colored according to their group identities obtained by k-means clustering. In Fig. 2b, we showed the PCA coefficient spectra whose x-axis and the y-axis show the Raman shifts and the PCA coefficients, respectively. We annotated the peak positions in this figure to relate the changes to molecular vibrations.

PCA coefficients are a measure of the variances between selected features. Since the first three PCs represent most of the spectra, we used them for spectral inspection. Here, we showed that the first PC is positively correlated with the peaks at 907, 1040 (Proline), 1247, 1265, 1657 (Amide III and Amide I), and 1442, 1295 (fatty acids, CH2/CH3). In Fig. 2e and f, we showed the spectra of proline and stearic acid to help the reader relate the differences in the wavenumbers to specific macromolecules. These correlations show that the first three principal component spectra are in line with the clustered Raman spectra shown in Fig. 2a. For example, the first group and the first PC have a common relation with the macromolecules proline and Amide I, which are the marker components of collagen. We showed the relations between the peak intensities at the strong peaks corresponding to major macromolecules in the first three PCs in Fig. 2d.

We concluded from Fig. 2d that the amplitude of the first PC spectrum at 1040 cm^-1^ is the highest while it is the lowest for the bands at 1295 and 1333 cm^-1^. Since the collagen structure is mainly composed of repeating proline chains, the difference between the signatures of other proteins and collagen structure in the tissue can be understood by following the relative changes of proline and Amide I bands^36^. The research on the preventive effect of polyunsaturated fatty acids on AF shows controversial outcomes^37–39^.

**Figure 3:**
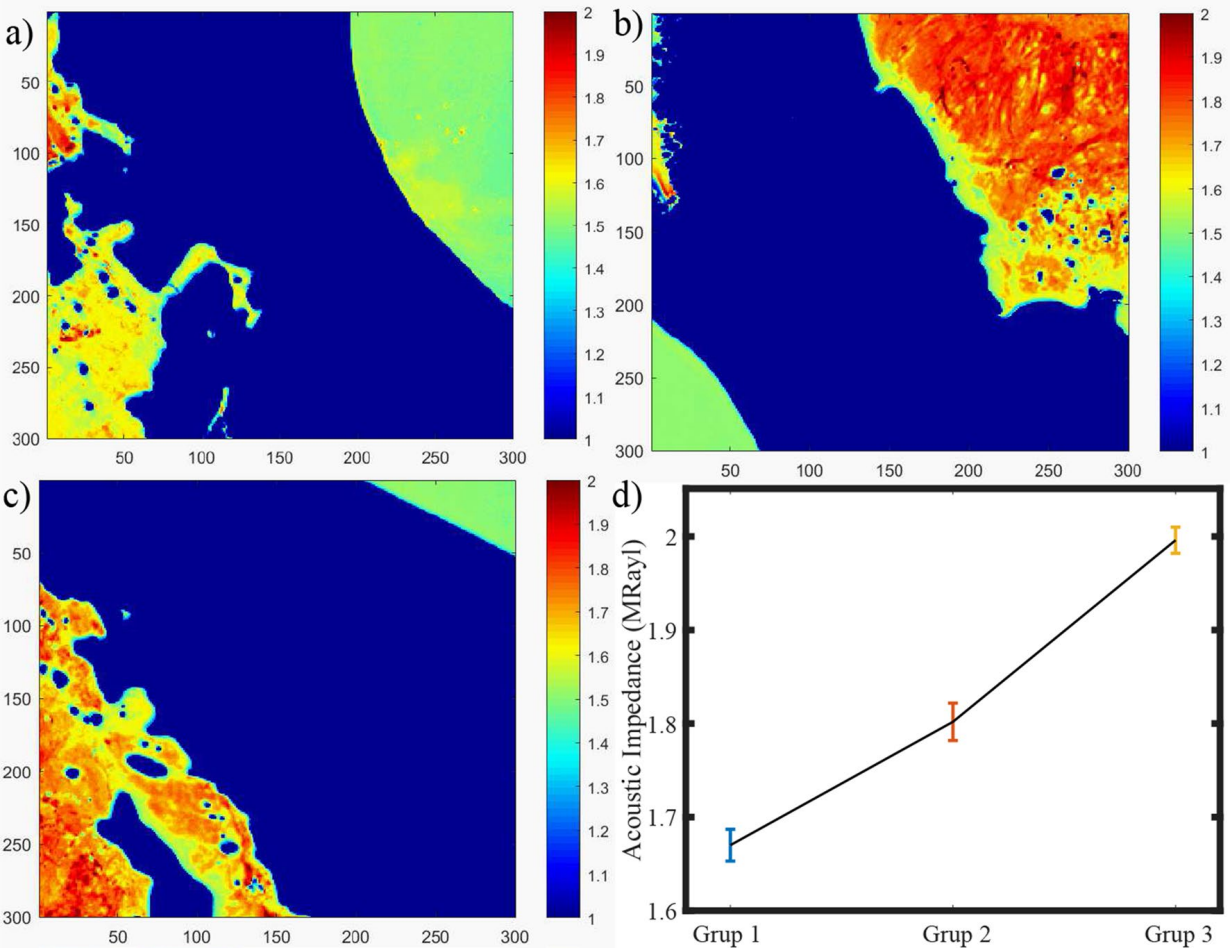
Differences in Raman shifts found by BCA. The box plot comparison for the peak locations located at (**a**) 817 cm^-1^, (**b**) 853 cm^-1^, (**c**) 938 cm^-1^, (**d**) 1060 cm^-1^, (**e**) 1120 cm^-1^, (**f**) 1438 cm^-1^ are displayed.

To extract more information from the Raman spectra, we quantified the Raman intensity differences between the groups visible in Fig. 2a. Therefore, we applied a Gaussian curve-fitting, also known as band component analysis (BCA), to decompose the measured Raman peaks into subcomponents that could not be resolved in the raw spectra. In this process, we calculated the parameters of the unresolved peak center positions, areas, and intensities and compared them using the indices we obtained from the k-means analysis. After determining peak parameters, we performed a Mann-Whitney U test on the peak intensities to find the statistically significant wavenumbers. Figure 3 shows the comparison of the Raman shift values of the peaks located at 817, 853, 938 cm^-1^ (proline and hydroxyproline), 1060, 1120, and 1438 (fatty acids) cm^-1^. Next, we calculated the means and standard deviations of the Raman intensities corresponding to the significant peaks to compare the groups. Finally, we summarized the average intensities of the significant peaks with their errors included in Table 1. Band component analysis showed that the second group has an apparent relationship with the fatty acid bands and the band intensities at collagen-related bands have the smallest values among the three groups. The band intensities at the statistically significant peak locations for the first and the third groups are closer to each other. One may speculate that the groups can be related to chronic AF, control, and postoperative AF when matched in order. Consequently, Raman analysis showed that the groups are different from each other in terms of the peak intensities corresponding to tissue cytoskeleton structures such as collagen.

**Table 1:** Significant Raman bands among calculated band component analysis and their tentative assignments.

Raman shift (cm^-1^)	Normalized Raman intensity (a.u.) *10^3^			Tentative assignments
	Group1	Group2	Group3	
817	9.2 ± 3.2	2.6 ± 5.3	7.2 ± 3.3	$\nu$(C-C) Protein backbone^29^
853	9.6 ± 4.7	3.6 ± 6.3	6.7 ± 4.8	Proline of collagen^30^
938	14.6 ± 5.2	1.7 ± 4.0	8.5 ± 3.4	C-H out-of-plane vibration (Collagen type I or tyrosine), $\nu$(C-C)^31,32^
1060	25.9 ± 6.7	70.9.1 ± 15.1	39.8 ± 8.3	$\nu$(C-C) Fatty acids^33^
1120	6.9 ± 0.4	39.4 ± 1.4	17.9 ± 1.4	$\nu$(C-C) Fatty acids^33^
1438	10.2 ± 8.0	59.1 ± 31.4	23.3 ± 13.4	HCC(out of plane), Proline^31^
1623	17.4 ± 1.4	18.7 ± 1.0	15.1 ± 1.4	Amide I $\beta$ sheet^34^
1676	6.8 ± 2.7	9.5 ± 3.1	8.7 ± 3.1	Amide I $\beta$ sheet^35^

### Scanning acoustic microscopy

**Figure 4:**
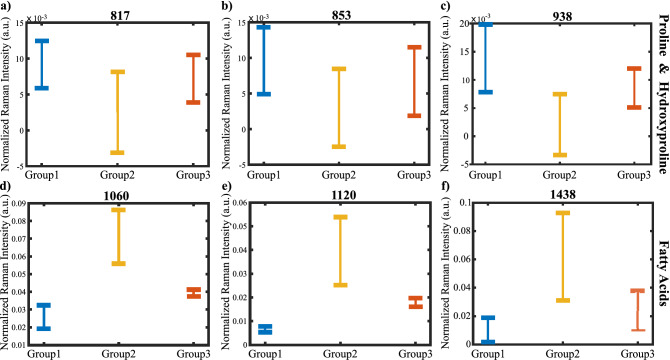
Acoustic impedance maps of tissues of patients of (**a**) Group 1, (**b**) Group 2, and (**c**) Group 3. (**d**) Average acoustic impedance values, with standard deviations, of 3 groups.

We obtained acoustic impedance maps of samples from 3 groups to characterize the mechanical properties of the tissues. Reflected ultrasound signals from the reference (distilled water) surface and the tissue sample surface on the polystyrene substrate constructed the maps (Fig. 4a). As can be seen in Fig. 4, the acoustic impedance map of the tissues from Group 3 shows higher values (Fig. 4c) when compared with the maps of the first (Fig. 4a) or the second group patients(Fig. 4b). We show in Fig. 4d, that the average acoustic impedance of the tissues from Group 1 is 1.648 ± 0.017 MRayl, while the average acoustic impedance of the second group is 1.783 ± 0.020 MRayl and the average acoustic impedance of the third group is 1.996 ± 0.014 MRayl. The average values of acoustic impedance were calculated considering the complete specimen surfaces.

As shown in Fig. 4d, the average acoustic impedance value was calculated to be the highest, indicating the highest elastic modulus, for Group 3, which may belong to patients with chronic AF. On the other hand, other groups had lower average acoustic impedance values, indicating lower elastic moduli. Therefore, samples of tissues of patients with chronic AF can be assumed to be firmer. In contrast, other tissues samples of patients who experienced post-operative AF and no AF can be assumed to be softer. Change in tissue structure and, therefore, tissue mechanics through aging, mechanical overload or injury is observed due to cardiac tissue damage, which is associated with the development of fibrosis. Furthermore, the presence of atrial fibrillation was found to be associated with arterial stiffness in patients with hypertension^40^. Conclusively, we can say that the success of discrimination of atrial tissue samples, with a lateral resolution of approximately $20\,\upmu \hbox {m}$ by SAM equipped with an 80 MHz transducer, is an indicator of the feasibility of this modality in the diagnosis of AF in clinics.

### Comparison of Raman and SAM measurements with the clinical labels

**Figure 5:**
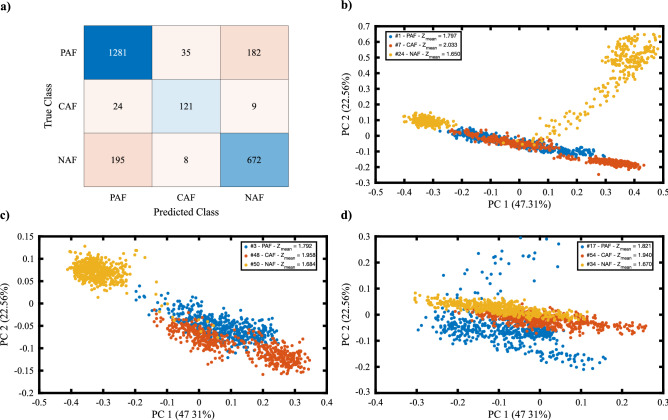
(**a**) Confusion table for the unsupervised neural network test set generated by using Raman data. (**b**–**d**) PCA score plot comparison plots. Each plot compares three clinical groups Legends includes the averaged acoustic impedance values. Abbreviations in the legend: CAF: Chornic AF, PAF: post operative AF, NAF: no AF.

We performed supervised classification using the clinical labels on Raman data. We applied neural networks classification after dividing the data 80 / 20% for training, cross-validation, and test purposes. We included each group obtained from k-means clustering in the dataset and calculated the accuracies. We found the best result for the dataset corresponding to Group 3. To provide a dataset representing similar biochemical profiles of the tissue and to make a consistent comparison, we included only one group in the training set. Figure 5a shows the confusion table for the test results obtained from Raman measurements. We found 82.1% test accuracy from the experiment results with Raman spectroscopy.

We selected only the tissue portion of the SAM images by applying k-means clustering to the data. We performed an ANOVA test on the cluster averaged acoustic impedances. Since we have only one feature, we did not use a similar training procedure for the SAM dataset. We found that the clinical labels are consistent with our acoustic impedance measurements with a confidence level of 99%. The biomechanical differences we measured with SAM are compatible with the biochemical profile of the group that gives us the best accuracy since the strong peaks in group 3 are originated from Amide I-III vibrations, most probably collagens.

We analyzed the PC scores to investigate the level of heterogeneity and the in-group / between-group similarity. In Fig. 5b–d, we show three examples of score plots generated for tissues from three clinically labeled groups. Although we have wide distribution for the same classes, the overlap between different classes is still enough to classify tissues from each other. Keeping in mind that we scanned a region whose dimensions are 1 mm X 1 mm, this level of heterogeneity is expected.

When we intersected the tissue predictions with Raman and SAM, we found a 67.5% match. Although the accuracy of Raman predictions and the level of confidence from the ANOVA test with SAM data is relatively high, we still have a modest matching. The reason might be the downsampling between Raman and SAM analysis. Since SAM generates one feature to analyze (acoustic impedance), we needed to find a score for every tissue from Raman spectral analysis. However, there are hundreds of variables (Rama shifts) in Raman measurements, which means the contribution to classification from each variable may be different, and it is challenging to find an unbiased parameter to compare. Converting the system into a multimodal microscope with a medical will enable simultaneous measurement and avoid such problems.

## Methods

### Ethics decleration

This study was ethically approved by Istanbul Medipol University Ethics Committee (Number: 2020/257) and informed consent was obtained from each participant. All experiments were performed in accordance with relevant guidelines and regulations.

### Surgical technique and material (tissue) retrieval

This study was ethically approved by Istanbul Medipol University Ethics Committee (Number: 2020/257) and informed consent was obtained from each participant. The operations were performed with standard median sternotomy. Cardiopulmonary bypass was instituted with aortic and two-stage atrial (for coronary artery bypass grafting or aortic valve procedures) or bicaval (for tricuspid and mitral valve procedures) cannulations after 3 mg/kg heparin infusion at activated clotting time above 480 seconds. The two-stage or superior caval vein cannula was inserted from the right atrial auricula and secured purse-string sutures. Patients were cooled down to 32 $^{\circ }$C, and planned surgical procedures were executed at moderate hypothermia on the arrested heart with a potassium enriched cold blood cardioplegia. Myocardial protection was achieved with intermittent cardioplegia infusion to the aortic root or directly into the coronary arteries. The cardiopulmonary bypass was finalized conventionally at the end of the planned interventions, and de-cannulation was performed. The tissue samples were obtained from the excluded right atrial auricula beyond the purse-string suture and immediately placed into 3% formaldehyde solution after neutralizing the heparin with protamin infusion (4.5 mg/kg). Operations were finalized with routine measures, and patients were transferred to the intensive care unit. Tissue samples were transferred to Bogazici University for microscopy studies.

### Raman spectroscopy

We built the customized experimental setup for Raman spectroscopy, as shown in Fig. 1a. We illuminated the sample with a diode laser whose wavelength was 785 nm (Laserglow). The beam was cleaned up with a line filter whose bandwidth was 10 nm and the output of the filter is transmitted through the dichroic mirror (Thorlabs SP805) and focused onto the sample plane after transmission from a 10X objective (Olympus, NA 0.25). The scattered beams were collected with the same optical path until the dichroic mirror. Since the scattered beams have the components of Stokes, Anti-Stokes, and Rayleigh beams, we selected Stokes photons, whose wavelengths are higher than the laser wavelength. These photons were reflected from the dichroic mirror and were steered into the achromatic lens that focuses the Raman photons into the multimode fiber (50 $\upmu \hbox {m}$, 0.22 NA, Thorlabs), which is coupled to the spectrometer (Ocean QE Pro Raman).

We performed the tissue measurements by scanning the tissue surface using a motorized stage (Standa). The stepsize of the scans was 50 $\mu$m and the scanning area was 1 mm x 1 mm. We acquired a total 441 spectra from each tissue. The integration time for the tissue measurements was two seconds. We applied a Boxcar averaging with a window size of one to smooth the spectrum in real-time. Finally, we applied a fluorescence baseline correction and vector normalization on the spectra described in our previous publications^14^. Combining these two pre-processing methods has already been discussed and found to be an efficient way to prepare data for spectral analysis^41^.

### Scanning acoustic microscope

Scanning acoustic microscope (AMS-50SI) developed by Honda Electronics (Toyohashi, Japan) was used for the characterization. SAM setup in acoustic impedance (AI) mode is shown in Fig. 1b. Ultrasonic signal generator and receiver in this study is an 80 MHz transducer. The focal length of the transducer is 1.5 mm and its spot size is 17 $\upmu \hbox {m}$. The coupling medium between the quartz lens and the substrate is distilled water. The ultrasonic signals generated by the transducer ($S_{0}$) are scanned 2-dimensionally by the X-Y stage and reflected back both from the reference ($S_{ref}$) and also the target material ($S_{target}$), then thsese reflected signals are compared and analyzed to generate the intensity and acoustic impedance maps of the region of interest with 300 x 300 sampling points with a lateral resolution of approximately 20 $\upmu \hbox {m}$^42^.

## Conclusion

AF is characterized by electrocardiogram which may lead to miss arrhythmia due to its duration of the measurement. Besides, tissue stiffness increases dramatically in tissues of patients with AF, too. Generally, the doctors diagnose this hardness by hand. However, we can quantify the estimation of stiffness in many other ways. Scanning acoustic microscopy is one of the most state-of-the-art and precise ways to do this. On the other hand, molecular changes that lead to mechanical changes in tissues can also be quantified by Raman spectroscopy. Combining these two techniques is a mechano-chemical analysis approach to assess the stiffness of the sample that can replace the subjective diagnosis done by medical doctors. We found increased acoustic impedance values for tissues of Groups 2 and 3, which can be related to AF development. Moreover, our Raman spectral analysis for these groups showed increased collagen levels, in agreement with the increase in stiffness.

There is a lack of literature on the diagnosis of AF by both SAM (no literature) and Raman spectroscopy (one study^5^). Therefore, we combined these two methods as a platform to assess the atrial tissues. Our limitation here was that we could not measure the tissues simultaneously by using both methods. This limitation is a challenge for us the overcome in future studies. Nevertheless, we think that the combination of SAM with RS has the potential to be used in clinics and will enable early diagnosis of AF in vivo with a verification. However, for combining SAM with RS, first, an intravascular SAM probe, similar to intravascular ultrasound (IVUS) probe, has to be developed. Then, it can be combined with RS on a catheter system for in vivo studies. Ultrasound signals and excitation light will be sent onto the tissue under investigation. Reflected ultrasound signals and Raman photons will be collected and then analyzed for obtaining affirmative morphological and mechanical information.

## Data Availability

The spectroscopy data that support the findings of this study are openly available in Zenodo at 10.5281/zenodo.5820544^43^.
